# *Chlamydia pecorum* Infection Associated with Ocular Disease in Goats in Alabama, USA

**DOI:** 10.3390/microorganisms13122715

**Published:** 2025-11-28

**Authors:** Jenna Workman Stockler, Thomas Passler, Anna-Catherine Bowden, Subarna Barua, Kelly Chenoweth, Chengming Wang

**Affiliations:** 1 Department of Clinical Sciences, College of Veterinary Medicine, Auburn University, Auburn, AL 36849, USA; jew0027@auburn.edu (J.W.S.); passlth@auburn.edu (T.P.); azb0261@auburn.edu (A.-C.B.); 2 Department of Pathobiology, College of Veterinary Medicine, Auburn University, Auburn, AL 36849, USA; szb0116@auburn.edu (S.B.); kjc0063@auburn.edu (K.C.); 3 Molecular Diagnostic Laboratory, College of Veterinary Medicine, Auburn University, Auburn, AL 36849, USA

**Keywords:** *Chlamydia pecorum*, goats, ocular infections, *Moraxella bovoculi*, USA

## Abstract

A herd of approximately 300 Spanish meat goats in central Alabama experienced sporadic ocular, respiratory, and reproductive diseases over two years, prompting diagnostic investigation at Auburn University’s JT Vaughan Large Animal Teaching Hospital. Five representative doelings exhibiting ocular lesions were examined. Clinical signs included conjunctivitis, corneal opacity, uveitis, and, in one severe case, systemic illness. Initial treatment with topical and systemic antibiotics provided incomplete resolution, raising suspicion of infectious keratoconjunctivitis of atypical etiology. Comprehensive diagnostic testing was performed, including aerobic and *Mycoplasma* cultures, Giemsa staining, and molecular assays. *Moraxella bovoculi* was cultured; however, Giemsa staining revealed *Chlamydia* elementary bodies, and a FRET-qPCR with DNA sequencing confirmed high *Chlamydia pecorum* loads (up to 1.1 × 10^7^ copies/swab). *Mycoplasma* testing was negative. Extended treatment with systemic and topical oxytetracycline led to gradual clinical improvement, with *C. pecorum* DNA declining over 22,000-fold and becoming undetectable after five weeks. This case represents the first documented report of *C. pecorum*–associated keratoconjunctivitis in goats in the United States. The findings underscore the diagnostic importance of molecular assays for detecting intracellular pathogens that may be missed by culture. The protracted treatment course highlights the therapeutic challenges posed by chlamydial infections due to their intracellular persistence. Additionally, the concurrent detection of *M. bovoculi* suggests the potential for mixed infections influencing disease severity. These results emphasize *C. pecorum* as an emerging pathogen of caprine ocular disease with implications for herd health and management.

## 1. Introduction

*Chlamydia pecorum* is an obligate intracellular bacterium that infects a wide range of ruminants worldwide, including cattle, sheep, and goats [1]. It has been implicated in polyarthritis, pneumonia, enteritis, conjunctivitis, and reproductive disorders such as abortion and infertility [1,2,3]. Although *C. pecorum* is well recognized as a pathogen of small ruminants in Australia, Europe and Asia [4,5,6,7,8,9], its prevalence and clinical impact in goats in the United States remain poorly defined. Ocular disease caused by *C. pecorum* is underreported, and uveitis attributable to this pathogen has rarely been described in small ruminants. *C. pecorum* traditionally causes surface ocular disease rather than intraocular disease with mucoid to mucopurulent ocular discharge, conjunctivitis characterized by significant chemosis, hyperemia, and follicle formation [10,11], and keratitis with corneal neovascularization and rare corneal ulceration. Most ocular cases are bilateral and symmetric [11].

Infectious keratoconjunctivitis (IKC) in goats is commonly attributed to *Mycoplasma* spp., especially *Mycoplasma conjunctivae*, with *Moraxella ovis* possibly contributing to ocular disease [12]. Treatment of these, typically extracellular, pathogens with standard topical or systemic antimicrobial therapy often results in rapid resolution. In contrast, the intracellular lifestyle of *C. pecorum* can complicate medical management, require extended treatment, and pose challenges to animal health and productivity.

This report describes an outbreak of ocular disease in Spanish goats in Alabama, with clinical manifestations ranging from conjunctivitis and keratitis to severe uveitis. Molecular diagnostics confirmed *C. pecorum* as the etiologic agent. One doeling required more than five weeks of systemic and topical antimicrobial treatment before bacterial resolution, underscoring the chronicity and therapeutic challenges of this infection. To our knowledge, this represents one of the first documented cases of *C. pecorum*-associated uveitis in goats in the United States.

## 2. Case Description

Auburn University’s JT Vaughan Large Animal Teaching Hospital (AULATH) was contacted by the herd owner of approximately 300 Spanish meat goats in central Alabama. Over two years, the owner had observed sporadic cases of ocular, respiratory, and reproductive disease. The goats were maintained on mixed summer grass pasture across two farms. FAMACHA scores were assessed monthly, and animals were dewormed as needed; no vaccines had been administered within the last four years. The owner reported recurrent episodes of “pinkeye,” characterized by epiphora to mucopurulent discharge, blepharospasm, and corneal opacity. Previous treatment attempts with topical triple antibiotic ointment (neomycin sulfate, polymyxin B, bacitracin) and systemic oxytetracycline provided incomplete resolution, although affected animals eventually improved. The owner also noted late-term abortions in breeding-age does and pneumonia in goats of various ages, although veterinary evaluation had not previously been sought.

Five approximately 10-month-old doelings with representative clinical signs were presented to AULATH. On admission, all animals were bright, alert, and responsive. Doelings #1 and #5 exhibited bilateral ocular discharge and conjunctivitis. Doeling #3 was febrile and had corneal edema with a pinpoint fluorescein-positive ulcer in the right eye. Doeling #2 had no ocular abnormalities. Doeling #4 was systemically ill, with dull mentation, poor body condition (1/5), tachypnea, increased bronchovesicular sounds, pale mucous membranes, and severe ocular lesions—specifically, a melting corneal ulcer in the right eye and uveitis in the left eye.

Based on the clinical presentation, infectious keratoconjunctivitis was suspected. Initial therapy included topical oxytetracycline/polymyxin B ointment (Terramycin^®^, Zoetis, Kalamazoo, MI, USA) every six hours. Doelings #3 and #4 also received IV flunixin meglumine (1.1 mg/kg q12h for 3 days) and topical autologous serum every six hours. Tulathromycin (5 mg/kg SQ, Draxxin^®^, Zoetis) was administered to all goats on day 3 due to persistence or progression of ocular lesions. Four doelings (#1, #2, #3, #5) improved sufficiently for discharge on day 8 with continued topical therapy.

**Figure 1:**
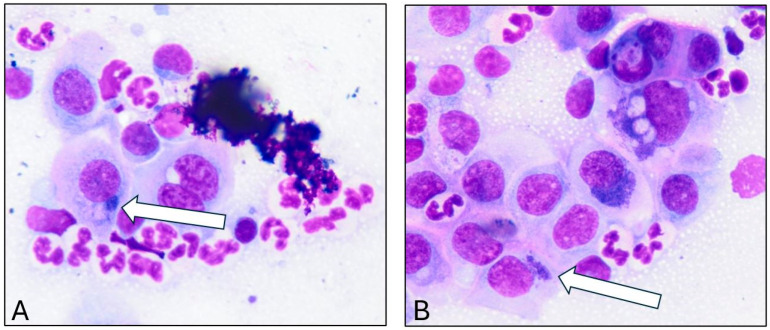
(**A**,**B**) **Elementary bodies within conjunctival epithelial cells after Giemsa staining.** The white arrows indicate the juxtanuclear, basophilic, intracytoplasmic inclusions typical of *Chlamydia* infection.

**Figure 2:**
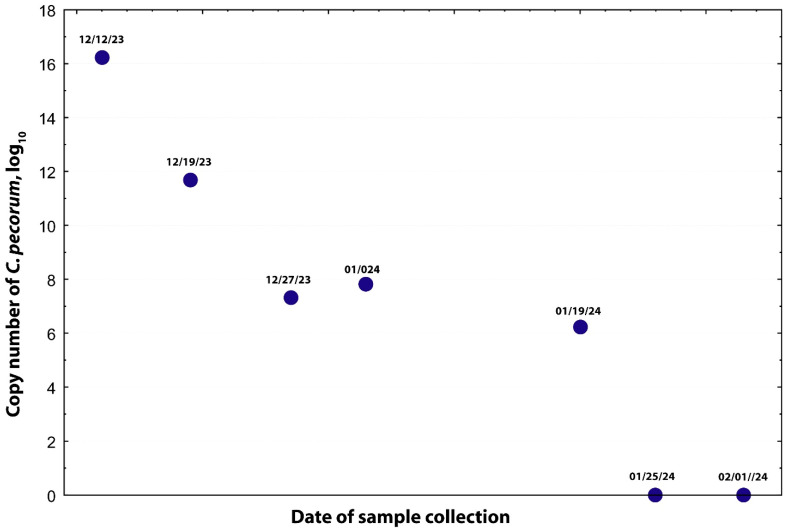
**Clearance of *C. pecorum* infection in a goat following prolonged oxytetracycline therapy**. Systemic oxytetracycline (22 mg/kg, subcutaneous, every 48 h) was administered for 11 doses (extra-label use) and discontinued to prevent potential toxicity. Topical oxytetracycline therapy was continued for an additional 27 days. To confirm microbial clearance, all antimicrobial treatments were withdrawn, and serial conjunctival swabs were analyzed by means of PCR. The initial *C. pecorum* load was 10^16.2^ copies per swab, which decreased by approximately 22,510-fold after five weeks of treatment.

Doeling #4 exhibited the most severe ocular involvement and was donated to AULATH for further diagnostic evaluation and treatment. Conjunctival swabs were submitted for aerobic culture after administration of topical anesthesia (proparacaine hydrochloride USP 0.5%, Alcon, Fort Worth, TX, USA), *Mycoplasma* culture, and PCR testing for *Chlamydia* and *Mycoplasma* at a commercial laboratory (Newport Laboratories—Vaxxinova), as well as for *Chlamydia*-specific PCR at the Molecular Diagnostic Laboratory, Auburn University College of Veterinary Medicine. *Moraxella bovoculi* was detected by the Newport Laboratory, while *Mycoplasma* testing was negative. Giemsa staining revealed abundant *Chlamydia* elementary bodies in conjunctival samples (Figure 1). FRET-qPCR followed by DNA sequencing and BLASTn as described [9,13], confirmed a high *C. pecorum* load, reaching up to 11,255,000 (10^16.2^) copies per swab (Figure 2).

Systemic oxytetracycline (22 mg/kg SQ q48h, Zoetis, Kalamazoo, MI, USA) was administered for 11 doses and discontinued due to potential for toxicity. Topical oxytetracycline therapy was continued for an additional 27 days. Extra-label drug use was performed with the consent of AULATH clinicians and complied with provisions of AMDUCA and 21 CFR 530. To confirm infection clearance, all antimicrobials were later withdrawn, and serial conjunctival PCR testing was performed (Figure 1). *C. pecorum* DNA levels decreased 22,510-fold following five weeks of treatment, and PCR results ultimately became negative. The doeling responded well and was discharged 120 days after initial hospitalization.

## 3. Discussion

This case highlights several clinically important aspects of *Chlamydia pecorum* infection in goats. Although *C. pecorum* has been well documented in ruminants globally, ocular disease—particularly keratoconjunctivitis—has rarely been reported in goats, and, to our knowledge, not previously in the United States. These findings emphasize the importance of considering *C. pecorum* in the differential diagnosis of caprine keratoconjunctivitis, alongside *Moraxella* and *Mycoplasma* spp. [14].

The protracted treatment required in this case illustrates the therapeutic challenges associated with chlamydial infections. The affected doeling required more than five weeks of systemic and topical antimicrobial therapy—considerably longer than typical regimens for infectious keratoconjunctivitis of other etiologies. This prolonged course likely reflects the intracellular persistence of *C. pecorum*, which limits antimicrobial penetration and complicates pathogen clearance. The use of molecular diagnostics was critical for confirming the etiologic agent and monitoring therapeutic response, as standard bacterial cultures would not have identified this intracellular organism. The elementary bodies on conjunctival cytology, while helpful to confirm suspicions in this case, may not be reliable in every case and appear in only one-third of cases [11].

At the herd level, the owner’s reports of abortions and respiratory disease may also be consistent with systemic or subclinical *C. pecorum* infections, given this pathogen’s broad tissue tropism. Although both *Moraxella bovoculi* and *C. pecorum* were detected, the clinical presentation, high chlamydial load, and treatment response strongly support *C. pecorum* as the primary cause of keratoconjunctivitis and uveitis in this case. Uveitis is a previously undocumented ocular sign of *C. pecorum* and may reflect breakdown of the blood-ocular barrier secondary to systemic inflammation. Nonetheless, the concurrent detection of *M. bovoculi* raises the possibility of mixed infections influencing disease expression or severity. Reports indicate that goats may be asymptomatic carriers of *Chlamydia* infections [6], and a 2024 study indicated *Mycoplasma conjunctivae*, a common co-infection with *Chlamydia*, can be cross-infected between livestock and wild ungulates [15]. Both wildlife and asymptomatic herd members could be sources of continued outbreaks. Future research is warranted to investigate the interactions between *M. bovoculi* and *C. pecorum*. Determination of whether co-infection exacerbates ocular inflammation or alters disease progression and treatment outcomes in goats and other ruminants is important for future directions.

Tulathromycin treatment appeared to be effective against *C. pecorum* infection in this study, likely due to its large volume of distribution and resulting high intracellular drug concentrations. In contrast, topically administered oxytetracycline achieves therapeutic levels only briefly and does not adequately penetrate the corneal or conjunctival tissues. Moreover, the cellular penetration of oxytetracycline is substantially lower than that of most macrolides and some fluoroquinolones. Overuse of oxytetracycline has also been associated with the development of tetracycline resistance in *Chlamydia suis* [16].

## 4. Conclusions

This case represents one of the first documented reports of *Chlamydia pecorum*–associated keratoconjunctivitis and uveitis in goats in the United States. It highlights the diagnostic value of molecular assays for accurate pathogen identification, the potential need for extended therapy, and the broader significance of *C. pecorum* as an emerging pathogen affecting the health and productivity of caprine.

## Data Availability

All data related to this work are included in the manuscript.
